# The Vascular Consequences of Metabolic Syndrome: Rodent Models, Endothelial Dysfunction, and Current Therapies

**DOI:** 10.3389/fphar.2020.00148

**Published:** 2020-03-04

**Authors:** Vivian Tran, T. Michael De Silva, Christopher G. Sobey, Kyungjoon Lim, Grant R. Drummond, Antony Vinh, Maria Jelinic

**Affiliations:** Department of Physiology, Anatomy and Microbiology, La Trobe University, Bundoora, VIC, Australia

**Keywords:** endothelial dysfunction, vascular disease, cardiometabolic abnormalities, nitric oxide, reactive oxygen species

## Abstract

Metabolic syndrome is characterized by visceral obesity, dyslipidemia, hyperglycemia and hypertension, and affects over one billion people. Independently, the components of metabolic syndrome each have the potential to affect the endothelium to cause vascular dysfunction and disrupt vascular homeostasis. Rodent models of metabolic syndrome have significantly advanced our understanding of this multifactorial condition. In this mini-review we compare the currently available rodent models of metabolic syndrome and consider their limitations. We also discuss the numerous mechanisms by which metabolic abnormalities cause endothelial dysfunction and highlight some common pathophysiologies including reduced nitric oxide production, increased reactive oxygen species and increased production of vasoconstrictors. Additionally, we explore some of the current therapeutics for the comorbidities of metabolic syndrome and consider how these benefit the vasculature.

## Overview

Metabolic syndrome is a growing epidemic affecting ~20% of adults (over a billion people) (O’Neill and O’Driscoll, 2015; Saklayen, 2018). This complex, multifactorial disorder arising from metabolic disturbances is characterized by visceral obesity, dyslipidemia, hyperglycemia and hypertension (Grundy et al., 2005). Another characteristic of metabolic syndrome is chronic low-grade inflammation (De Ferranti and Mozaffarian, 2008; Sharma, 2011). These factors all contribute to the elevated risk of cardiovascular disease, acute cardiovascular events (including stroke and myocardial infarction), type 2 diabetes mellitus (T2DM), or further complications such as renal disease (O’Neill and O’Driscoll, 2015; Tune et al., 2017). Vascular dysfunction is a key contributor to the pathogenesis of all of these disorders (Rajendran et al., 2013). Metabolic syndrome not only causes social and economic burdens, but significantly impacts morbidity and mortality. This review will describe how metabolic syndrome affects the regulation of vascular function and tone. Specifically, we will focus on rodent models of metabolic syndrome, highlighting the changes that occur to endothelial function and adipose tissue and consider relevance to clinical translation in humans. We will also discuss potential areas for further research to advance knowledge on vascular pathophysiology in metabolic syndrome.

### Current Rodent Models for Metabolic Syndrome

An ideal translational animal model for metabolic syndrome would closely resemble the human anatomy and pathophysiology of the disease (Emini Veseli et al., 2017). Thus, an important consideration when choosing an animal model is that it mimics the key clinical criteria that define metabolic syndrome. The International Diabetes Federation defines metabolic syndrome as central obesity and at least two of the following: dyslipidemia (>150 mg/dl plasma triglycerides and/or reduced high-density lipoproteins (HDL) < 40 mg/dl for men and <50 mg/dl for women), elevated blood pressure (≥130 mmHg systolic and/or ≥ 85 mmHg diastolic), or hyperglycemia (≥100 mg/dl fasting plasma glucose) (Alberti et al., 2006). Importantly, many patients do not present with all of these classifications, and similarly there is no one animal model that mimics all of these abnormalities of metabolic syndrome.

#### Genetic Mouse Models

Genetic models of obesity and diabetes allow for the evaluation of specific molecular mechanisms. C57BL/6J-*Lep^ob^* mice, more commonly known as *ob/ob* mice, were one of the first genetic models of obesity. These mice lack leptin due to a spontaneous homozygous mutation on the leptin gene, causing marked obesity, hyperinsulinemia, and hyperglycemia by 12 weeks of age. By approximately 24 weeks of age, *ob/ob* mice develop left ventricular hypertrophy and cardiac fibrosis and are in a pro-inflammatory state (La Cava, 2017). The C57BL/KsJ-db/db (*db/db*) mouse is a related genetic mouse model, which has a defective leptin receptor (Wang et al., 2014). By 13 weeks of age, *db/db* mice are overweight and have hyperglycemia and dyslipidemia (increased plasma triglycerides, total cholesterol, and non-esterified fatty acids). Importantly, endothelium-dependent aortic relaxation to acetylcholine (ACh) is impaired whereas that to direct nitric oxide donors remains unaffected, indicating endothelial dysfunction (Dong et al., 2010). Additionally, *db/db* mice have elevated circulating leptin which promotes a pro-inflammatory state, linked to the increased activity of interleukin-6 (IL-6) (La Cava, 2017). Neither *ob/ob* nor *db/db* mice, however, display increased blood pressure—unlike a large proportion of humans with metabolic syndrome—and are therefore not ideal models for the many such people with metabolic syndrome (Mark et al., 1999).

#### Genetic Rat Models

Zucker Fatty rats are among the most common genetic rat models of metabolic syndrome and are deficient in the leptin receptor due to a missense mutation in the gene. This increases circulating leptin levels and rats are obese by 3–5 weeks of age (Aleixandre de Artinano and Miguel Castro, 2009). These rats variably develop hyperglycemia (the severity is variable between studies, and sometimes within the same cohort), dyslipidemia, and hypertension (Zucker and Zucker, 1961; Marsh et al., 2007; Yokoi et al., 2013; Wang et al., 2014; Wong et al., 2016). However, several studies also report conflicting data, with lower systolic blood pressure in Zucker fatty rats compared to the lean controls (Aleixandre de Artinano and Miguel Castro, 2009). Thus, while in some studies the model does appear to accurately reflect the presentation of metabolic syndrome patients in the clinic, inconsistencies between different studies make it difficult to develop definitive conclusions.

The Dahl salt-sensitive rat is widely used to study salt-induced hypertension and, when crossed with Zucker fatty rats, the resulting offspring are DahlS.Z-Lepr^fa^/Lepr^fa^ (DS/obese) rats. DS/obese rats have hyperphagia and develop abdominal obesity, hypertension, dyslipidemia, and T2DM and thus, appear to be a useful model for advanced metabolic syndrome (Hattori et al., 2011). Obese spontaneously hypertensive rats (also known as Koletsky rats) are another animal model used to study metabolic syndrome. These rats are obese by 5 weeks of age and develop hyperlipidemia even when fed a normal chow diet. Mild hyperinsulinemia is present with only slight hyperglycemia. At 3 months of age, spontaneous hypertension occurs with mean arterial pressure rising to ≥180 mmHg (Aleixandre de Artinano and Miguel Castro, 2009).

#### Diet-Induced Rodent Models

Diet modifications are often used to study metabolic syndrome due to pronounced effects on metabolism and in turn, hormonal, glucose, and lipid pathways. Fructose-enriched diets are effective for inducing metabolic syndrome and act *via* several mechanisms to promote obesity (Johnson et al., 2007). Mechanisms relevant to the satiety center suggest that fructose stimulates the production of insulin and leptin but inhibits ghrelin (Teff et al., 2004). Other studies suggest that the addition of fructose simply makes food more appetizing and stimulates increased food intake and weight gain (Lowette et al., 2015). Simple and complex carbohydrates are essential nutrients and the main source of energy for the body. Adopting a sedentary lifestyle in conjunction with excessive carbohydrate consumption can result in an imbalance in energy, which increases blood glucose and increases release of insulin. This imbalance predisposes individuals to weight gain and decreases insulin sensitivity (Wong et al., 2016).

A high fat diet (HFD) can also be used to induce metabolic syndrome. Mice fed a HFD from 4 to 6 weeks of age develop obesity, hyperglycemia, and endothelial dysfunction after 10 weeks (Kobayasi et al., 2010; Liu et al., 2016). In some instances, systolic blood pressure is mildly raised (by ~10 mmHg), suggesting a pre-hypertensive state (Taylor et al., 2018). HFD mice have increased quantities of white adipose tissue, which enhances the expression of pro-inflammatory mediators such as tumor necrosis factor alpha (TNF-α). This mechanism is thought to be a key driver for insulin resistance in obesity (Makki et al., 2013). To date, numerous types of HFD regimens have been used, with variations in the amount of fat (20 to 60% of total energy) and its source (lard, beef tallow, olive, or coconut oil) as well as the duration of feeding and age of animals. The fat source appears to be particularly important. Fats derived from lard, coconut and olive oil increase body weight, plasma insulin and triglyceride and decrease plasma adiponectin concentrations in male Wistar rats (Buettner et al., 2006). Alternatively, beef tallow derived-fat increases plasma leptin, insulin, and lipid concentrations (Hsu et al., 2009).

HFD rodent models display most of the features of metabolic syndrome, but patients with metabolic syndrome would typically consume a higher proportion of simple carbohydrates than most HFD models in the literature (Panchal and Brown, 2011). Diets comprising both high fat and high carbohydrate components promote even more of the features of metabolic syndrome in rodents and are therefore more clinically representative than just HFD alone (Panchal and Brown, 2011). One potential criticism of these diet-induced models is that they rarely lead to atherosclerosis. Thus, HFD regimens are often combined with mice that are genetically dyslipidemic to incorporate the atherosclerotic phenotype in metabolic syndrome. For example, apolipoprotein E-deficient (*ApoE^−/−^*) mice and low density lipoprotein receptor deficient (*LDLR^−/−^*) mice show similar metabolic profiles to the diet-induced models described above, but have the added complication of advanced atherosclerosis (Emini Veseli et al., 2017).

Despite there being a variety of rodent models of metabolic syndrome available (summarized in **Table 1**), the precise mechanisms behind the progression to a diseased vascular state remain poorly understood. Obesity and the abnormalities associated with metabolic syndrome (i.e., hypertension, dyslipidemia, hyperglycemia) adversely impact vascular structure and function (Beckman et al., 2002). The remainder of this review will address this.

**Table 1:** Summary of different rodent models of metabolic syndrome and their effects on varying vessels.

Model	Age	Species	Sex	Vessel	Effect of metabolic syndrome	Ref
HFD (45% kcal from fat) for 32 weeks	37 weeks	C57BL/6J mice	M	MA	↑ Superoxide and NOX activity in PVAT	(Gil-Ortega et al., 2014)
WD (30% fructose, 20% lard, 18% protein, 5% cellulose) for 42 weeks	50 weeks	Sprague-Dawley rats	M	TA	↑ROCK pathway associated with insulin resistance	(Elrashidy et al., 2019)
High carbohydrate, HFD (% kcal from fat + 15% fructose in drinking water)	24 weeks	Sprague-Dawley rats	M	CA, MA	↑Insulin sensitivity and lipid profiles; ↓SBP	(Senaphan et al., 2015)
HFD (59% kcal from fat) for 16 weeks	24 weeks	Swiss mice	F	Aorta	↑ SBP and DBP; ↓ aortic relaxation to ACh but not SNP; ⟷ aortic IL-1β and IL-6 protein expression; ↓ aortic NF-kB	(Kobayasi et al., 2010)
HFD (42% kcal from fat) for 30 weeks	35 weeks	C57BL/6J mice	M	TA, CA	↑Prostanoids and vascular thromboxane receptor gene expression	(Traupe et al., 2002)
C57BL/6J-*Lep^ob^ (ob/ob)*	27–32 weeks	C57BL/6J mice	M	Aorta MA	↑Plasma insulin, PKC activity, GRK2 protein levels; ↓aortic insulin-induced relaxation, ACh-induced relaxation	(Winters et al., 2000; Taguchi et al., 2011)
C57BL/KsJ-db/db (*db/db*)	16 weeks	C57BL/KsJ mice	M	MA	↑Production of superoxide anions; ↓ACh-induced relaxation and BH_4_ bioavailability	(Pannirselvam et al., 2002)
Zucker diabetic fatty (ZDF *fa/fa*) rat	9–11 weeks	Zucker diabetic fatty rats	M	Aorta	↑FFA-induced NADPH oxidase activation and ROS production	(Chinen et al., 2007)
Spontaneously hypertensive rats	14 months	Spontaneously hypertensive rats	M	TA	↑ROS formation, NADPH oxidase activity and protein expression of NOX 1 and NOX 2; ↓ACh-induced relaxation	(Wind et al., 2010)
HFD (20.5% protein, 35.7% carbohydrates, and 36.0% fat)	24–28 weeks	Dahl-Salt Sensitive rats	F and M	Aorta	↑HFD male and female SBP at 4 weeks and CD4+ T cells and T helper cells, greater CD3+ T cells in males, and greater % of pro-inflammatory T cells in males	(Taylor et al., 2018)

ACh, acetylcholine; BH_4_, tetrahydrobiopterin; CA, carotid arteries; DBP, diastolic blood pressure; F, female; FFA, free fatty acid; GRK2, G protein-coupled receptor kinase 2; HFD, high fat diet; IL, interleukin; Kcal, kilocalorie; M, male; MA, mesenteric arteries; NF-kB, nuclear factor kappa beta; NOX, NADPH oxidase; PKC, protein kinase C; PVAT, perivascular adipose tissue; ROCK, Rho kinase; SBP, systolic blood pressure; SNP, sodium nitroprusside; TA, thoracic aorta; WD, western diet.

### The Role of Metabolic Syndrome Comorbidities on Endothelial Dysfunction

**Figure 1:**
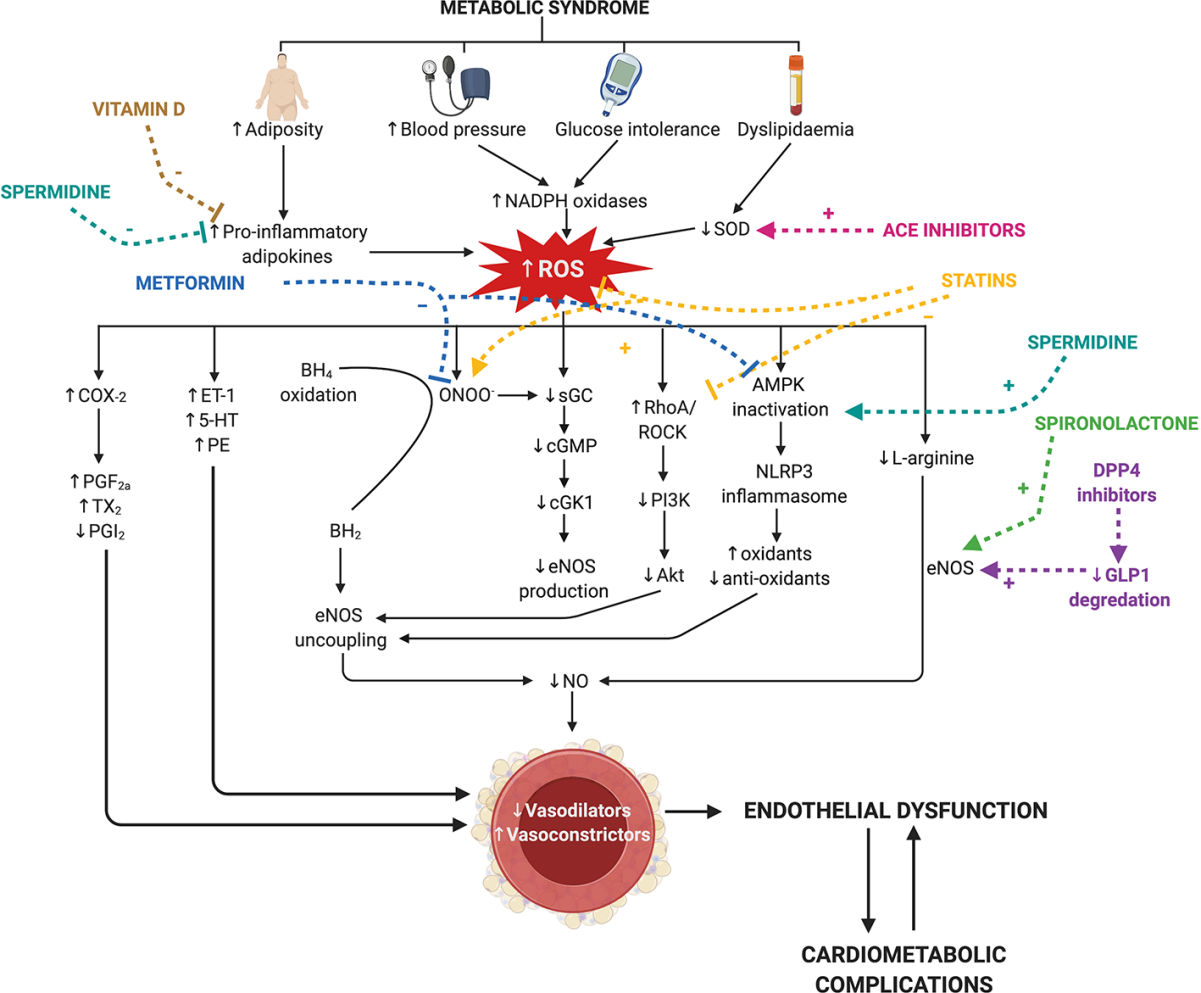
Current therapies for the comorbidities of metabolic syndrome, targetting nitric oxide and reactive oxygen species signaling in endothelial dysfunction. Metabolic syndrome is characterized by an increase in visceral adiposity, blood pressure, glucose intolerance, and dyslipidemia. Individually, these co-morbidities induce endothelial dysfunction by increasing reactive oxygen species (ROS) and reducing nitric oxide (NO; pathways indicated in black). ROS is increased *via* increases in nicotinamide adenine dinucleotide phosphate (NADPH) oxidase and pro-inflammatory adipokines and reductions in superoxide dismutase (SOD). This reduces endothelial nitric oxide synthase (eNOS) production *via* two key mechanisms: reduced L-arginine conversion and soluble guanylate cyclase (sGC) activity. Uncoupling of eNOS occurs *via* two mechanisms [tetrahydrobiopterin (BH_4_) and 5′-AMP-activated protein kinase (AMPK) inactivation] to further reduce eNOS activity. Increased cyclooxygenase-2 (COX-2) activity drives the production of vasoconstrictor prostanoids (PGF_2a_, prostaglandin F2α; TXA_2_, thromboxane A2) and decreases prostacyclin (PGI_2_) production. ROS also drives the production of other endothelium-derived contracting factors (ET-1= endothelin-1, 5-HT= serotonin and PE= phenylephrine). Many first-line therapeutic drugs for the co-morbidities of metabolic syndrome (colored) target these mechanisms. Metformin (blue) reduces AMPK inactivation and peroxynitrite (ONOO-) production. Angiotensin converting enzyme (ACE) inhibitors (pink) reduce SOD activity. Statins (yellow) reduce AMPK inactivation and ROS production and increase sGC activity. Spironolactone and dipeptidyl peptidase-4 (DPP4) increase eNOS activity. Spermidine (turquoise) and vitamin D (dark yellow) inhibit the activation of pro-inflammatory adipokines released from adipose tissue, and spermidine promotes AMPK activation. BH_2_, 7,8-dihydrobiopterin; cGMP, cyclic guanosine-3′,5′-monophosphate; cGK1, cGMP-dependent protein kinase-1; ROCK, RhoA associated protein kinase; GLP1, glucagon-like peptide 1. Created with BioRender.com.

Endothelial dysfunction predisposes the vasculature to a heightened contractile state due to an imbalance between endothelium-derived relaxing (e.g., NO, PGI_2_, EDH downregulation) and contracting factors (e.g., TxA_2_, ET-1 upregulation) (Guzik et al., 2000; Lerman and Zeiher, 2005). Endothelial dysfunction also promotes pro-inflammatory and oxidative stress pathways *via* endothelial mitochondrial reactive oxygen species (ROS) driving vascular growth and remodeling (Cai and Harrison, 2000; Shenouda et al., 2011; Widlansky and Gutterman, 2011). This fundamental switch of the endothelium in metabolic syndrome to a dysfunctional state, involves the host immune system and production of ROS (Deanfield et al., 2007), and the progression of diseases occurs *via* a variety of dynamic changes within the vasculature (**Figure 1**). There are many detailed reviews regarding the function of the endothelium in a physiological state (Cai and Harrison, 2000; Endemann and Schiffrin, 2004; Hadi et al., 2005; Rajendran et al., 2013), and thus, this review will focus on the mechanisms of endothelial dysfunction that accompany the comorbidities of metabolic syndrome. Some animal models of metabolic syndrome inherently present with multiple comorbidities—for example diet-induced models may present hyperglycemia, dyslipidemia, and obesity. However, the studies mentioned in this section focus on individual comorbidities and their effect on endothelial dysfunction.

#### Hyperglycemia

In *db/db and ob/ob* mice, endothelium-dependent vasodilatation is impaired in the coronary arterioles, aorta, and mesenteric arteries. This impairment is associated with enhanced superoxide production and the activation of immune responses downstream of the NACHT, LRR, and PYD domains-containing protein 3 inflammasome, which reduces the function of endothelium-dependent relaxing factors and the regulation of insulin (Bagi et al., 2003; Okon et al., 2003; Vandanmagsar et al., 2011). Endothelial dysfunction is not only a consequence of insulin resistance, but also impairs insulin signaling to further reduce insulin sensitivity, thereby resulting in a destructive cycle in metabolic syndrome and diabetes. In obese Zucker rats, altered insulin signaling disrupts insulin-mediated NO production (*via* downregulation of eNOS expression) to impair vasodilatation in resistance arteries. The involvement of ROS and subsequent degradation of BH_4_ (a cofactor essential for NO synthesis from eNOS) synthesis in insulin resistance is thought to play a role in the impairment of NO-dependent vasodilatation (Eringa et al., 2007). In T2DM patients, ROS reduces the availability of BH_4_ (Heitzer et al., 2000). Reduced interaction between BH_4_ and eNOS leads to eNOS uncoupling and production of superoxide instead of NO (Heitzer et al., 2000). In that study, an infusion of BH_4_ partially counteracted the reduced ACh-induced vasodilation, demonstrating that eNOS uncoupling and reduction of NO availability contribute to endothelial dysfunction in T2DM (Heitzer et al., 2000). An early study using female streptozotocin (STZ)-induced diabetic rats found impaired endothelial function in mesenteric arteries due mainly to altered production of vasodilators rather than ROS (Taylor et al., 1992). Endothelial dysfunction is region-specific in this model— as endothelial impairment was absent in the aortae of the diabetic animals (Taylor et al., 1994). The therapeutic potential of antioxidants has been a key area of interest in hyperglycemia research due to their ability to scavenge/neutralize ROS (Morrow et al., 2003; Davis et al., 2006; Versari et al., 2009). However, large clinical studies have investigated the effects of anti-oxidant vitamins (such as vitamin E and C) in diabetes, and these did not reduce the incidence of vascular disease (Heart Protection Study Collaborative G, 2002; Xu and Zou, 2009). Furthermore, acute hyperglycemia promotes vasoconstrictor-prostanoid production and thus, an increased vascular smooth muscle cells (VSMC) contractility and vascular tone (Bagi et al., 2003; Okon et al., 2003).

There are a number of therapeutics available for the treatment of hyperglycemia. Pharmacological therapies such as thiazolidinediones, statins, and metformin not only improve insulin sensitivity, but also endothelium-dependent vasodilation in patients with type 2 diabetes (Paniagua et al., 2002; Yee A et al., 2004; Xu and Zou, 2009) and in diabetic rodent studies (Kanda M and Ichihara, 2003; Wong et al., 2006). Metformin is the first-line drug used for the treatment of hyperglycemia. Despite this, the precise mechanisms by which metformin lowers blood glucose levels are still unclear, but AMPK activation is thought to be a key target of action (Eriksson and Nystrom, 2014). AMPK is also thought to be a potential target in reversing endothelial dysfunction by promoting eNOS phosphorylation to stimulate NO production (Davis et al., 2006; Xu and Zou, 2009). Conversely, cell-culture studies indicate that this occurs independently of AMPK activation in mouse microvascular endothelial cells, but rather *via* eNOS and Akt phosphorylation (Ghosh et al., 2015). Thus, the involvement of AMPK in metformin therapy may require the involvement of other cell-types. A newer therapeutic strategy for hyperglycemia is glucagon‐like peptide‐1 receptor agonists and dipeptidyl peptidase‐4 inhibitors. Glucagon-like peptide-1 is a direct endothelium-dependent vasodilator, and is also NOS-dependent. The vasoprotective effects of glucagon-like peptide-1 receptor agonists have been shown in a number of clinical studies too, however, there are also studies that show detrimental effects on the vasculature (Ban et al., 2008). Such detrimental effects appear to occur with chronic long-term administration (>4 weeks), highlighting the importance of determining long-term effects of hyperglycemia medications on the vasculature.

#### Dyslipidemia

Endothelial relaxation is impaired *via* multiple pathways in dyslipidemia. In humans with hypercholesterolemia, ACh-induced vasodilatation is reduced, whereas G_i_-independent bradykinin-induced vasodilatation remains unchanged (Matsumoto et al., 2004; Gendron et al., 2007).This indicates a selective loss of some vasorelaxation pathways in hypercholesterolemia (Matsumoto et al., 2004; Gendron et al., 2007). Rodent models have been previously used to study lipid metabolism and its links to cardiovascular disease, however there are significant differences between lipid metabolism in rodents and humans. For example, mice carry the majority of plasma cholesterol in HDL, whereas humans carry it in low-density lipoproteins (LDL) (Gordon et al., 2015). However, there are similarities between the species that should be noted. For the protein diversity of HDL and LDL size ranges are similar in both humans and mice, and mice have minor proteins that are identified in humans which play a role in inflammation and innate immunity (Gordon et al., 2015). The majority of rodent dyslipidemia studies to date (characterized by increased triglycerides, decreased HDL and abnormal LDL) have been performed in ApoE^−/−^ mice. Interestingly, despite severe hypercholesterolemia, young ApoE^−/−^ mice have normal vascular function. Importantly, once challenged with either HFD or ageing to induce atherosclerotic lesions, relaxation (both endothelial-dependent and -independent) is impaired.

In non-rodent animal models, such as hypercholesterolemic rabbits, L-arginine treatment inhibits atherosclerosis and improves NO-mediated vasodilatation in thoracic and abdominal aortae and iliac arteries by enhanced NO synthesis and eNOS expression (Jeremy et al., 1996; Hayashi et al., 2005) The increased levels of oxidized low-density LDL in dyslipidemia has cytotoxic potential and atherogenic properties, and may also attenuate NO activity. In cultured human endothelial cells, oxidized LDL exposure decreased eNOS messenger RNA (mRNA) expression (Shi et al., 2014). In human umbilical vein endothelial cells, eNOS mRNA degradation is also linked to upregulation of the pro-inflammatory cytokine TNF-α in atherosclerotic lesions. Therefore, pro-inflammatory cytokines that interfere with eNOS mRNA levels may reduce eNOS activity and impair vasorelaxation in dyslipidemia (Yoshizumi et al., 1993). Transmembrane receptor LOX-1 can also directly mediate oxidized LDL inducing superoxide formation through the activation of nuclear factor kappa B (Cominacini et al., 2000; Sangle and Shen, 2010). Not only does this contribute to lipid accumulation through macrophages and inflammatory cytokines, high levels of circulating oxidized LDL acts on receptors that decrease L-arginine availability, thus altering NO production and ultimately endothelial function (Saraswathi and Hasty, 2006). Statins are used in the clinic to lower blood LDL cholesterol. An added benefit of statins is that they also reverse endothelial dysfunction in dyslipidemic patients (Bonetti et al., 2003). Cell culture studies confirm that statins stabilize eNOS mRNA to increase NO production in human endothelial cells (Bonetti et al., 2003). Additionally, statins λ also reduce nicotinamide adenine dinucleotide phosphate (NADPH) oxidase activity by downregulating NOX-1 mRNA expression, thus suppressing O_2_^−^ generation in hypertensive rats further contributing to the protective effects of statins (Wassmann et al., 2002; Antonopoulos et al., 2012). Spermidine, a precursor to polyamines, has been shown to exert anti-inflammatory properties, and to inhibit age-related oxidative protein damage and ROS. To target lipid metabolism, spermidine induces AMPK pathway to regulate autophagy, in turn inhibiting expression of fatty acids (Gao et al., 2018).

#### Obesity

In obesity, the severity of endothelial dysfunction strongly correlates with the degree of visceral adiposity (Lobato et al., 2012). This is likely to be due to multiple pathways, such as adipocyte hypertrophy, hypoxia and macrophage infiltration (Leal Vde and Mafra, 2013). Similar to hypertensive conditions, visceral adiposity increases oxidative stress, and promotes changes in the pro-inflammatory adipokine profile resulting in eNOS uncoupling (Li et al., 2015). Specifically, circulating pro-inflammatory adipokines such as visfastin, apelin, retinol binding protein-4, vaspin, serum amyloid A, plasminogen activator inhibitor-1, angiotensinogen, chemerin and are increased in obesity. Conversely, obesity is associated with reduced adiponectin, omentin, and zinc-α2-glycoprotein (Leal Vde and Mafra, 2013). In obesity, NADPH oxidase upregulation also accentuates ROS production and induce endothelial dysfunction in the aorta (Serpillon et al., 2009; Jiang et al., 2011). In endothelial cells, the nuclear factor-κB (NF-κB) pathway mediates inflammation by increasing ROS production and reducing NO production (Kobayasi et al., 2010). Additionally, surrounding adipocytes secrete products that stimulate the increase of adhesion molecules and apoptosis of endothelial cells (Kobayasi et al., 2010). Although obese patients present with elevated NF-κB expression, it is unknown whether the direct inhibition of this pathway improves endothelium-dependent relaxation (Silver et al., 2007). Weight loss is the primary objective for obese patients. In obese patients with essential hypertension, calorie restriction demonstrated beneficial effects and improvement in endothelium-dependent vasodilation stimulating an increased release of nitric oxide (Sasaki et al., 2002).

In diet-induced obese mice, vascular dysfunction (in the thoracic aorta and carotid artery) is associated with increased thromboxane gene expression and vasoconstrictor prostanoids (Traupe et al., 2002). Non-selective COX inhibition blocks ACh-induced contraction but selective inhibition of COX-2 is without effect (Traupe et al., 2002). Additionally, thromboxane synthase inhibitors did not affect ACh-induced contraction, indicating that vascular dysfunction in obesity is driven by upregulation of vascular thromboxane receptor and endothelium-dependent prostanoid vasoconstrictors (Traupe et al., 2002). COX-inhibition also altered ET-1-induced contraction (Traupe et al., 2002; Mundy et al., 2007). This provides evidence that, not surprisingly, multiple mechanisms are involved in endothelial dysfunction in obese rodents. Importantly though, diet-induced obese mice are normotensive, indicating that obesity-induced endothelial dysfunction is likely independent of changes to blood pressure.

Epidemiological studies indicate that low vitamin D levels are associated with all of aforementioned co-morbidities of metabolic syndrome (Snijder et al., 2005; Awad et al., 2012). *In vitro* studies demonstrate that vitamin D3 inhibits pre-adipocyte proliferation by downregulating adipogenesis genes (Zhuang et al., 2007) and reducing obesity-induced inflammation (Marcotorchino et al., 2012). Despite ample evidence that vitamin D hinders the development of adipose, the precise mechanism by which vitamin D influences obesity has not yet been elucidated.

#### Hypertension

The pathophysiology of hypertension is multifactorial and related to activation of the sympathetic nervous system, renin-angiotensin-aldosterone system, pro-inflammatory mediators, endothelial dysfunction, and increased oxidative stress (Oparil et al., 2003). Sustained elevated pressure in the vasculature promotes premature ageing and increased endothelial cell turnover (Bleakley et al., 2015). The regenerated endothelial cells have an impaired ability to release endothelium-derived relaxing factors (Tang and Vanhoutte, 2010). Endothelial dysfunction has been demonstrated in most animal models of hypertension including spontaneously hypertensive rats (SHRs), angiotensin II-induced hypertension, Dahl salt-sensitive rats, and the two-kidney one-clip model (Stankevicius et al., 2002; Yang et al., 2004; Michel et al., 2008). A sustained elevation of blood pressure is linked with decreased levels of NO and increased vascular ROS (Konukoglu and Uzun, 2017). Oxidative stress plays a major role in the pathophysiology of hypertension-induced endothelial dysfunction. ROS alone promote vasoconstriction and impair antioxidant production (Santilli et al., 2015). Superoxide and other ROS inhibit NO bioavailability in several ways. Superoxide can react directly with NO to form peroxynitrite. This leads to eNOS uncoupling, thus aggravating the reduced NO production and promoting endothelial dysfunction (Bakker et al., 2009). Peroxynitrite can also nitrate other proteins, altering their function (Pacher et al., 2007). This correlates with studies in hypertensive patients reporting decreased NO availability and increased serum malondialdehyde [a clinical indicator of elevated ROS; (Wattanapitayakul et al., 2000; Guzik et al., 2002; Armas-Padilla et al., 2007)]. Increased NADPH oxidase activity has been observed in angiotensin II-induced hypertension, deoxycorticosterone acetate-salt hypertension and SHRs. In angiotensin II-infused mice, increased ROS is linked to eNOS uncoupling, BH_4_ oxidation and further increases in superoxide, impairing endothelial function. This is also associated with downregulation of downstream targets of NO, such as cyclic-GMP, soluble guanylate cyclase, protein kinase G-dependent phosphorylation, S-nitrosylation, and transnitrosylation (Mollnau et al., 2002; Zhang, 2017). Clinically, there is an abundance of pharmacological treatments for hypertension that directly target the renin angiotensin aldosterone system (incl. angiotensin converting enzyme inhibitors and angiotensin II receptor blockers). In addition to blocking renal sodium reabsorption and plasma volume expansion (Ferrario and Schiffrin, 2015), many of these also improve endothelial dysfunction. This occurs *via* inhibition of vascular angiotensin I and II conversion and by increasing NO bioavailability (Farquharson and Struthers, 2000). The precise mechanisms by which this occurs varies between the different types of drugs. Mineralocorticoid receptor antagonists such as spironolactone increases NO bioavailability *via* the upregulation of eNOS and downregulation of the proinflammatory cytokine TGF-ß (Adel et al., 2014). ACE inhibitors increase NO bioavailability *via* three key mechanisms: increased intracellular calcium to increase NO production; blocking natural endopeptidase to inhibit local bradykinin degradation; and enhancing activity of the antioxidant superoxide dismutase (Enseleit et al., 2003).

The transformation of arachidonic acid by cyclooxygenase results in the production of endoperoxides, releasing endothelial-derived contracting factors (Vanhoutte et al., 2005). Importantly, many rodent studies show evidence of increased vasoconstrictor prostanoid responses in hypertension (Vanhoutte et al., 2005; Vanhoutte and Tang, 2008). Conversely, blunted endothelium-dependent vasodilation is the key underlying cause of vascular dysfunction in hypertensive humans (Vanhoutte et al., 2005). Therefore, while the impact of hypertension on the vasculature is similar between species, the underlying mechanisms may differ. This highlights the challenge of translating pre-clinical findings to a clinical setting. Thus, identification and use of the most representative animal models of human disease are vital for progressing our understanding of these conditions.

## Conclusion

Accompanying the global rise in obesity, metabolic syndrome is an escalating public health concern. Metabolic syndrome is a multifactorial disorder, and hence it is not surprising that numerous signaling pathways contribute to the subsequent endothelial dysfunction. Despite this, the majority of current therapies that treat the comorbidities of metabolic syndrome and improve endothelial dysfunction target NO and ROS signaling (**Figure 1**). Future studies should investigate the effects of therapeutics which target vasoconstrictor prostanoids, another key mechanism of endothelial dysfunction in metabolic syndrome.

## Author Contributions

VT, MJ and AV wrote the manuscript. VT and MJ created figure and table. All authors contributed to the planning and drafting of the review.

## Conflict of Interest

The authors declare that the research was conducted in the absence of any commercial or financial relationships that could be construed as a potential conflict of interest.

The handling editor declared a shared affiliation, though no other collaboration, with one of the authors MJ at time of review.

## References

[B1] Adel H. Taye A. Khalifa M. M. (2014). Spironolactone improves endothelial dysfunction in streptozotocin-induced diabetic rats. Naunyn Schmiedebergs Arch. Pharmacol. 387 (12), 1187–1197. 10.1007/s00210-014-1048-3 25238812

[B2] Alberti K. G. Zimmet P. Shaw J. (2006). Metabolic syndrome: a new world-wide definition. a consensus statement from the international diabetes federation. Diabetes Med. 23 (5), 469–480. 10.1111/j.1464-5491.2006.01858.x 16681555

[B3] Aleixandre de Artinano A. Miguel Castro M. (2009). Experimental rat models to study the metabolic syndrome. Br. J. Nutr. 102 (9), 1246–1253. 10.1017/S0007114509990729 19631025

[B4] Antonopoulos A. S. Margaritis M. Shirodaria C. Antoniades C. (2012). Translating the effects of statins: from redox regulation to suppression of vascular wall inflammation. Thromb. Haemost. 108 (5), 840–848. 10.1160/TH12-05-0337 22872079

[B5] Armas-Padilla M. C. Armas-Hernandez M. J. Sosa-Canache B. Cammarata R. Pacheco B. Guerrero J. (2007). Nitric oxide and malondialdehyde in human hypertension. Am. J. Ther. 14 (2), 172–176. 10.1097/01.pap.0000249914.75895.48 17414586

[B6] Awad A. B. Alappat L. Valerio M. (2012). Vitamin d and metabolic syndrome risk factors: evidence and mechanisms. Crit. Rev. Food Sci. Nutr. 52 (2), 103–112. 10.1080/10408391003785458 22059957

[B7] Bagi Z. Koller A. Kaley G. (2003). Superoxide-NO interaction decreases flow- and agonist-induced dilations of coronary arterioles in Type 2 diabetes mellitus. Am. J. Physiol. Heart Circ. Physiol. 285 (4), 1404–1410. 10.1152/ajpheart.00235.2003 12805026

[B8] Bakker W. Eringa E. C. Sipkema P. van Hinsbergh V. W. (2009). Endothelial dysfunction and diabetes: roles of hyperglycemia, impaired insulin signaling and obesity. Cell Tissue Res. 335 (1), 165–189. 10.1007/s00441-008-0685-6 18941783

[B9] Ban K. Noyan-Ashraf M. H. Hoefer J. Bolz S. S. Drucker D. J. Husain M. (2008). Cardioprotective and vasodilatory actions of glucagon-like peptide 1 receptor are mediated through both glucagon-like peptide 1 receptor-dependent and -independent pathways. Circulation 117 (18), 2340–2350. 10.1161/CIRCULATIONAHA.107.739938 18427132

[B10] Beckman J. A. Creager M. A. Libby P. (2002). Diabetes and atherosclerosis: epidemiology, pathophysiology, and management. JAMA 287 (19), 2570–2581. 10.1001/jama.287.19.2570 12020339

[B11] Bleakley C. Hamilton P. K. Pumb R. Harbinson M. McVeigh G. E. (2015). endothelial function in hypertension: victim or culprit? J. Clin. Hypertens. 17 (8), 651–654. 10.1111/jch.12546 PMC8031772 25857326

[B12] Bonetti P. O. Lerman L. O. Napoli C. Lerman A. (2003). Statin effects beyond lipid lowering–are they clinically relevant? Eur. Heart J. 24 (3), 225–248. 10.1016/S0195-668X(02)00419-0 12590901

[B13] Buettner R. Parhofer K. G. Woenckhaus M. Wrede C. E. Kunz-Schughart L. A. Scholmerich J. (2006). Defining high-fat-diet rat models: metabolic and molecular effects of different fat types. J. Mol. Endocrinol. 36 (3), 485–501. 10.1677/jme.1.01909 16720718

[B14] Cai H. Harrison D. G. (2000). Endothelial dysfunction in cardiovascular diseases: the role of oxidant stress. Circ. Res. 87 (10), 840–844. 10.1161/01.RES.87.10.840 11073878

[B15] Chinen I. Shimabukuro M. Yamakawa K. Higa N. Matsuzaki T. Noguchi K. (2007). Vascular lipotoxicity: endothelial dysfunction *via* fatty-acid-induced reactive oxygen species overproduction in obese zucker diabetic fatty rats. Endocrinology 148 (1), 160–165. 10.1210/en.2006-1132 17023526

[B16] Cominacini L. Pasini A. F. Garbin U. Davoli A. Tosetti M. L. Campagnola M. (2000). Oxidized low density lipoprotein (ox-LDL) binding to ox-LDL receptor-1 in endothelial cells induces the activation of NF-kappaB through an increased production of intracellular reactive oxygen species. J. Biol. Chem. 275 (17), 12633–12638. 10.1074/jbc.275.17.12633 10777555

[B17] Davis B. J. Xie Z. Viollet B. Zou M. H. (2006). Activation of the AMP-activated kinase by antidiabetes drug metformin stimulates nitric oxide synthesis *in vivo* by promoting the association of heat shock protein 90 and endothelial nitric oxide synthase. Diabetes 55 (2), 496–505. 10.2337/diabetes.55.02.06.db05-1064 16443786

[B18] De Ferranti S. Mozaffarian D. (2008). The perfect storm: obesity, adipocyte dysfunction, and metabolic consequences. Clin. Chem. 54 (6), 945–955. 10.1373/clinchem.2007.100156 18436717

[B19] Deanfield J. E. Halcox J. P. Rabelink T. J. (2007). Endothelial function and dysfunction: testing and clinical relevance. Circulation 115 (10), 1285–1295. 10.1161/CIRCULATIONAHA.106.652859 17353456

[B20] Dong Y. F. Liu L. Kataoka K. Nakamura T. Fukuda M. Tokutomi Y. (2010). Aliskiren prevents cardiovascular complications and pancreatic injury in a mouse model of obesity and type 2 diabetes. Diabetologia 53 (1), 180–191. 10.1007/s00125-009-1575-5 19894030

[B21] Elrashidy R. A. Zhang J. Liu G. (2019). Long-term consumption of western diet contributes to endothelial dysfunction and aortic remodeling in rats: implication of Rho-kinase signaling. Clin. Exp. Hypertens. 41 (2), 174–180. 10.1080/10641963.2018.1462375 29667441

[B22] Emini Veseli B. Perrotta P. De Meyer G. R. A. Roth L. Van der Donckt C. Martinet W. (2017). Animal models of atherosclerosis. Eur. J. Pharmacol. 816, 3–13. 10.1016/j.ejphar.2017.05.010 28483459

[B23] Endemann D. H. Schiffrin E. L. (2004). Endothelial dysfunction. J. Am. Soc. Nephrol. 15 (8), 1983–1992. 10.1097/01.ASN.0000132474.50966.DA 15284284

[B24] Enseleit F. Lüscher T. F. Ruschitzka F. (2003). Angiotensin-converting enzyme inhibition and endothelial dysfunction: focus on ramipril. Eur. Heart J. Suppl. 5 (suppl_A), A31–AA6. 10.1016/S1520-765X(03)90061-7

[B25] Eriksson L. Nystrom T. (2014). Activation of AMP-activated protein kinase by metformin protects human coronary artery endothelial cells against diabetic lipoapoptosis. Cardiovasc. Diabetol. 13, 1–9 152. 10.1186/s12933-014-0152-5 25391818 PMC4234893

[B26] Eringa E. C. Stehouwer C. D. Roos M. H. Westerhof N. Sipkema P. (2007). Selective resistance to vasoactive effects of insulin in muscle resistance arteries of obese Zucker (fa/fa) rats. Am. J. Physiol. Endocrinol. Metab. 293 (5), 1134–1139. 10.1152/ajpendo.00516.2006 17623751

[B27] Farquharson C. A. Struthers A. D. (2000). Spironolactone increases nitric oxide bioactivity, improves endothelial vasodilator dysfunction, and suppresses vascular angiotensin I/angiotensin II conversion in patients with chronic heart failure. Circulation 101 (6), 594–597. 10.1161/01.CIR.101.6.594 10673249

[B28] Ferrario C. M. Schiffrin E. L. (2015). Role of mineralocorticoid receptor antagonists in cardiovascular disease. Circ. Res. 116 (1), 206–213. 10.1161/CIRCRESAHA.116.302706 25552697 PMC4283558

[B29] Gao M. Zhao W. Li C. Xie X. Li M. Bi Y. (2018). Spermidine ameliorates non-alcoholic fatty liver disease through regulating lipid metabolism *via* AMPK. Biochem. Biophys. Res. Commun. 505 (1), 93–98. 10.1016/j.bbrc.2018.09.078 30241944

[B30] Gendron M. E. Thorin-Trescases N. Villeneuve L. Thorin E. (2007). Aging associated with mild dyslipidemia reveals that COX-2 preserves dilation despite endothelial dysfunction. Am. J. Physiol. Heart Circ. Physiol. 292 (1), 451–458. 10.1152/ajpheart.00551.2006 16980343

[B31] Ghosh S. Lakshmanan A. P. Hwang M. J. Kubba H. Mushannen A. Triggle C. R. (2015). Metformin improves endothelial function in aortic tissue and microvascular endothelial cells subjected to diabetic hyperglycaemic conditions. Biochem. Pharmacol. 98 (3), 412–421. 10.1016/j.bcp.2015.10.008 26467186

[B32] Gil-Ortega M. Condezo-Hoyos L. Garcia-Prieto C. F. Arribas S. M. Gonzalez M. C. Aranguez I. (2014). Imbalance between pro and anti-oxidant mechanisms in perivascular adipose tissue aggravates long-term high-fat diet-derived endothelial dysfunction. PloS One 9 (4), 953. 10.1371/journal.pone.0095312 PMC3997398 24760053

[B33] Gordon S. M. Li H. Zhu X. Shah A. S. Lu L. J. Davidson W. S. (2015). A comparison of the mouse and human lipoproteome: suitability of the mouse model for studies of human lipoproteins. J. Proteome Res. 14 (6), 2686–2695. 10.1021/acs.jproteome.5b00213 25894274 PMC4712022

[B34] Grundy S. M. Cleeman J. I. Daniels S. R. Donato K. A. Eckel R. H. Franklin B. A. (2005). Diagnosis and management of the metabolic syndrome: an american heart association/national heart, lung, and blood institute scientific statement. Circ 112 (17), 2735–2752. 10.1161/CIRCULATIONAHA.105.169404 16157765

[B35] Guzik T. J. West N. E. Black E. McDonald D. Ratnatunga C. Pillai R. (2000). Vascular superoxide production by NAD(P)H oxidase: association with endothelial dysfunction and clinical risk factors. Circ. Res. 86 (9), 85–90. 10.1161/01.RES.86.9.e85 10807876

[B36] Guzik T. J. West N. E. Pillai R. Taggart D. P. Channon K. M. (2002). Nitric oxide modulates superoxide release and peroxynitrite formation in human blood vessels. Hypertension 39 (6), 1088–1094. 10.1161/01.HYP.0000018041.48432.B5 12052847

[B37] Hadi H. A. Carr C. S. Al Suwaidi J. (2005). Endothelial dysfunction: cardiovascular risk factors, therapy, and outcome. Vasc. Health Risk Manage. 1 (3), 183–198. PMC1993955 17319104

[B38] Hattori T. Murase T. Ohtake M. Inoue T. Tsukamoto H. Takatsu M. (2011). Characterization of a new animal model of metabolic syndrome: the DahlS.Z-Lepr(fa)/Lepr(fa) rat. Nutr. Diabetes 1, e1–e6. 10.1038/nutd.2010.1 23154293 PMC3302131

[B39] Hayashi T. Juliet P. A. Matsui-Hirai H. Miyazaki A. Fukatsu A. Funami J. (2005). l-Citrulline and l-arginine supplementation retards the progression of high-cholesterol-diet-induced atherosclerosis in rabbits. Proc. Natl. Acad. Sci. U. S. A 102 (38), 13681–13686. 10.1073/pnas.0506595102 16157883 PMC1224660

[B40] Heart Protection Study Collaborative G (2002). MRC/BHF heart protection study of antioxidant vitamin supplementation in 20,536 high-risk individuals: a randomised placebo-controlled trial. Lancet 360 (9326), 23–33. 10.1016/S0140-6736(02)09328-5 12114037

[B41] Heitzer T. Krohn K. Albers S. Meinertz T. (2000). Tetrahydrobiopterin improves endothelium-dependent vasodilation by increasing nitric oxide activity in patients with type ii diabetes mellitus. Diabetologia 43 (11), 1435–1438. 10.1007/s001250051551 11126415

[B42] Hsu C. L. Wu C. H. Huang S. L. Yen G. C. (2009). Phenolic compounds rutin and o-coumaric acid ameliorate obesity induced by high-fat diet in rats. J. Agric. Food Chem. 57 (2), 425–431. 10.1021/jf802715t 19119847

[B43] Jeremy R. W. McCarron H. Sullivan D. (1996). Effects of dietary L-arginine on atherosclerosis and endothelium-dependent vasodilatation in the hypercholesterolemic rabbit. response according to treatment duration, anatomic site, and sex. Circulation 94 (3), 498–506. 10.1161/01.cir.94.3.498 8759095

[B44] Jiang F. Lim H. K. Morris M. J. Prior L. Velkoska E. Wu X. (2011). Systemic upregulation of NADPH oxidase in diet-induced obesity in rats. Redox Rep. 16 (6), 223–229. 10.1179/174329211X13049558293713 22195989 PMC6837396

[B45] Johnson R. J. Segal M. S. Sautin Y. Nakagawa T. Feig D. I. Kang D. H. (2007). Potential role of sugar (fructose) in the epidemic of hypertension, obesity and the metabolic syndrome, diabetes, kidney disease, and cardiovascular disease. Am. J. Clin. Nutr. 86 (4), 899–906. 10.1093/ajcn/86.4.899 17921363

[B46] Kanda M S. K. Ichihara K. (2003). Effects of atorvastatin and pravastatin on glucose tolerance in diabetic rats mildly induced by streptozotocin. Biol. Pharm. Bull. 26 (12), 1681–1684. 10.1248/bpb.26.1681 14646170

[B47] Kobayasi R. Akamine E. H. Davel A. P. Rodrigues M. A. Carvalho C. R. Rossoni L. V. (2010). Oxidative stress and inflammatory mediators contribute to endothelial dysfunction in high-fat diet-induced obesity in mice. J. Hypertens. 28 (10), 2111–2119. 10.1097/HJH.0b013e32833ca68c 20616756

[B48] Konukoglu D. Uzun H. (2017). Endothelial dysfunction and hypertension. Adv. Exp. Med. Biol. 956, 511–540. 10.1007/5584_2016_90 28035582

[B49] La Cava A. (2017). Leptin in inflammation and autoimmunity. Cytokine 98, 51–58. 10.1016/j.cyto.2016.10.011 27916613 PMC5453851

[B50] Leal Vde O. Mafra D. (2013). Adipokines in obesity. Clin. Chim Acta 419, 87–94. 10.1016/j.cca.2013.02.003 23422739

[B51] Lerman A. Zeiher A. M. (2005). Endothelial function: cardiac events. Circulation 111 (3), 363–368. 10.1161/01.CIR.0000153339.27064.14 15668353

[B52] Li Q. Youn J. Y. Cai H. (2015). Mechanisms and consequences of endothelial nitric oxide synthase dysfunction in hypertension. J. Hypertens. 33 (6), 1128–1136. 10.1097/HJH.0000000000000587 25882860 PMC4816601

[B53] Liu C. Chang C. Lee H. Chen Y. Tsai T. Chiang Chiau J. (2016). Metabolic damage presents differently in young and early-aged C57BL/6 mice fed a high-fat diet. Int. J. Gerontol. 10 (2), 105–111. 10.1016/j.ijge.2015.10.004

[B54] Lobato N. S. Filgueira F. P. Akamine E. H. Tostes R. C. Carvalho M. H. Fortes Z. B. (2012). Mechanisms of endothelial dysfunction in obesity-associated hypertension. Braz. J. Med. Biol. Res. 45 (5), 392–400. 10.1590/S0100-879X2012007500058 22488221 PMC3854291

[B55] Lowette K. Roosen L. Tack J. Vanden Berghe P. (2015). Effects of high-fructose diets on central appetite signaling and cognitive function. Front. Nutr. 2, 1–5. 10.3389/fnut.2015.00005 25988134 PMC4429636

[B56] Makki K. Froguel P. Wolowczuk I. (2013). Adipose tissue in obesity-related inflammation and insulin resistance: cells, cytokines, and chemokines. ISRN Inflamm. 2013, 139–239. 10.1155/2013/139239 PMC3881510 24455420

[B57] Marcotorchino J. Gouranton E. Romier B. Tourniaire F. Astier J. Malezet C. (2012). Vitamin D reduces the inflammatory response and restores glucose uptake in adipocytes. Mol. Nutr. Food Res. 56 (12), 1771–1782. 10.1002/mnfr.201200383 23065818

[B58] Mark A. L. Shaffer R. A. Correia M. L. Morgan D. A. Sigmund C. D. Haynes W. G. (1999). Contrasting blood pressure effects of obesity in leptin-deficient ob/ob mice and agouti yellow obese mice. J. Hypertens. 17 (12), 1949–1953. 10.1097/00004872-199917121-00026 10703894

[B59] Marsh S. A. Powell P. C. Agarwal A. Dell’Italia L. J. Chatham J. C. (2007). Cardiovascular dysfunction in zucker obese and zucker diabetic fatty rats: role of hydronephrosis. Am. J. Physiol. Heart Circ. Physiol. 293 (1), 292–298. 10.1152/ajpheart.01362.2006 17351065

[B60] Matsumoto T. Sato A. Suenaga H. Kobayashi T. Kamata K. (2004). Modulations of shear stress-induced contractile responses and agonist-induced vasodilation in hypercholesterolemic rats. Atherosclerosis 175 (1), 31–38. 10.1016/j.atherosclerosis.2004.02.017 15186944

[B61] Michel F. S. Man G. S. Man R. Y. Vanhoutte P. M. (2008). Hypertension and the absence of EDHF-mediated responses favour endothelium-dependent contractions in renal arteries of the rat. Br. J. Pharmacol. 155 (2), 217–226. 10.1038/bjp.2008.256 18574459 PMC2538696

[B62] Mollnau H. Wendt M. Szocs K. Lassegue B. Schulz E. Oelze M. (2002). Effects of angiotensin II infusion on the expression and function of NAD(P)H oxidase and components of nitric oxide/cGMP signaling. Circ. Res. 90 (4), E58–E65. 10.1161/01.RES.0000012569.55432.02 11884382

[B63] Morrow V. A. Foufelle F. Connell J. M. Petrie J. R. Gould G. W. Salt I. P. (2003). Direct activation of AMP-activated protein kinase stimulates nitric-oxide synthesis in human aortic endothelial cells. J. Biol. Chem. 278 (34), 31629–31639. 10.1074/jbc.M212831200 12791703

[B64] Mundy A. L. Haas E. Bhattacharya I. Widmer C. C. Kretz M. Baumann K. (2007). Endothelin stimulates vascular hydroxyl radical formation: effect of obesity. Am. J. Physiol. Regul. Integr. Comp. Physiol. 293 (6), 2218–2224. 10.1152/ajpregu.00295.2007 17898123

[B65] O’Neill S. O’Driscoll L. (2015). Metabolic syndrome: a closer look at the growing epidemic and its associated pathologies. Obes Rev. 16 (1), 1–12. 10.1111/obr.12229 25407540

[B66] Okon E. B. Szado T. Laher I. McManus B. van Breemen C. (2003). Augmented contractile response of vascular smooth muscle in a diabetic mouse model. J. Vasc. Res. 40 (6), 520–530. 10.1159/000075238 14646372

[B67] Oparil S. Zaman M. A. Calhoun D. A. (2003). Pathogenesis of hypertension. Ann. Intern Med. 139 (9), 761–776. 10.7326/0003-4819-139-9-200311040-00011 14597461

[B68] Pacher P. Beckman J. S. Liaudet L. (2007). Nitric oxide and peroxynitrite in health and disease. Physiol. Rev. 87 (1), 315–424. 10.1152/physrev.00029.2006 17237348 PMC2248324

[B69] Panchal S. K. Brown L. (2011). Rodent models for metabolic syndrome research. J. BioMed. Biotechnol. 2011, 351–982. 10.1155/2011/351982 PMC3018657 21253582

[B70] Paniagua J. A. Lopez-Miranda J. Escribano A. Berral F. J. Marin C. Bravo D. (2002). Cerivastatin improves insulin sensitivity and insulin secretion in early-state obese type 2 diabetes. Diabetes 51 (8), 2596–2603. 10.2337/diabetes.51.8.2596 12145176

[B71] Pannirselvam M. Verma S. Anderson T. J. Triggle C. R. (2002). Cellular basis of endothelial dysfunction in small mesenteric arteries from spontaneously diabetic (db/db -/-) mice: role of decreased tetrahydrobiopterin bioavailability. Br. J. Pharmacol. 136 (2), 255–263. 10.1038/sj.bjp.0704683 12010774 PMC1573335

[B72] Rajendran P. Rengarajan T. Thangavel J. Nishigaki Y. Sakthisekaran D. Sethi G. (2013). The vascular endothelium and human diseases. Int. J. Biol. Sci. 9 (10), 1057–1069. 10.7150/ijbs.7502 24250251 PMC3831119

[B73] Saklayen M. G. (2018). The global epidemic of the metabolic syndrome. Curr. Hypertens. Rep. 20 (2), 1–8. 10.1007/s11906-018-0812-z 29480368 PMC5866840

[B74] Sangle G. V. Shen G. X. (2010). Signaling mechanisms for oxidized LDL-induced oxidative stress and the upregulation of plasminogen activator inhibitor-1 in vascular cells. Clin. Lipidology. 5 (2), 221–232. 10.2217/clp.10.6

[B75] Santilli F. D’Ardes D. Davi G. (2015). Oxidative stress in chronic vascular disease: from prediction to prevention. Vascul Pharmacol. 74, 23–37. 10.1016/j.vph.2015.09.003 26363473

[B76] Saraswathi V. Hasty A. H. (2006). The role of lipolysis in mediating the proinflammatory effects of very low density lipoproteins in mouse peritoneal macrophages. J. Lipid Res. 47 (7), 1406–1415. 10.1194/jlr.M600159-JLR200 16639077

[B77] Sasaki S. Higashi Y. Nakagawa K. Kimura M. Noma K. Sasaki S. (2002). A low-calorie diet improves endothelium-dependent vasodilation in obese patients with essential hypertension. Am. J. Hypertens. 15 (4), 302–309. 10.1016/S0895-7061(01)02322-6 11991214

[B78] Senaphan K. Kukongviriyapan U. Sangartit W. Pakdeechote P. Pannangpetch P. Prachaney P. (2015). Ferulic acid alleviates changes in a rat model of metabolic syndrome induced by high-carbohydrate, high-fat diet. Nutrients 7 (8), 6446–6464. 10.3390/nu7085283 26247970 PMC4555122

[B79] Serpillon S. Floyd B. C. Gupte R. S. George S. Kozicky M. Neito V. (2009). Superoxide production by NAD(P)H oxidase and mitochondria is increased in genetically obese and hyperglycemic rat heart and aorta before the development of cardiac dysfunction. The role of glucose-6-phosphate dehydrogenase-derived NADPH. Am. J. Physiol. Heart Circ. Physiol. 297 (1), 153–162. 10.1152/ajpheart.01142.2008 PMC2711743 19429815

[B80] Sharma P. (2011). Inflammation and the metabolic syndrome. Indian J. Clin. Biochem. 26 (4), 317–318. 10.1007/s12291-011-0175-6 23024465 PMC3210244

[B81] Shenouda S. M. Widlansky M. E. Chen K. Xu G. Holbrook M. Tabit C. E. (2011). Altered mitochondrial dynamics contributes to endothelial dysfunction in diabetes mellitus. Circulation 124 (4), 444–453. 10.1161/CIRCULATIONAHA.110.014506 21747057 PMC3149100

[B82] Shi Y. Luscher T. F. Camici G. G. (2014). Dual role of endothelial nitric oxide synthase in oxidized LDL-induced, p66Shc-mediated oxidative stress in cultured human endothelial cells. PloS One 9 (9), e107787. 10.1371/journal.pone.0107787 25247687 PMC4172699

[B83] Silver A. E. Beske S. D. Christou D. D. Donato A. J. Moreau K. L. Eskurza I. (2007). Overweight and obese humans demonstrate increased vascular endothelial NAD(P)H oxidase-p47(phox) expression and evidence of endothelial oxidative stress. Circulation 115 (5), 627–637. 10.1161/CIRCULATIONAHA.106.657486 17242275

[B84] Snijder M. B. van Dam R. M. Visser M. Deeg D. J. Dekker J. M. Bouter L. M. (2005). Adiposity in relation to vitamin D status and parathyroid hormone levels: a population-based study in older men and women. J. Clin. Endocrinol. Metab. 90 (7), 4119–4123. 10.1210/jc.2005-0216 15855256

[B85] Stankevicius E. Martinez A. C. Mulvany M. J. Simonsen U. (2002). Blunted acetylcholine relaxation and nitric oxide release in arteries from renal hypertensive rats. J. Hypertens. 20 (8), 1571–1579. 10.1097/00004872-200208000-00020 12172319

[B86] Taguchi K. Kobayashi T. Matsumoto T. Kamata K. (2011). Dysfunction of endothelium-dependent relaxation to insulin *via* PKC-mediated GRK2/Akt activation in aortas of ob/ob mice. Am. J. Physiol. Heart Circ. Physiol. 301 (2), H571–H583. 10.1152/ajpheart.01189.2010 21572010

[B87] Tang E. H. Vanhoutte P. M. (2010). Endothelial dysfunction: a strategic target in the treatment of hypertension? Pflugers Arch. 459 (6), 995–1004. 10.1007/s00424-010-0786-4 20127126

[B88] Taylor P. D. McCarthy A. L. Thomas C. R. Poston L. (1992). Endothelium-dependent relaxation and noradrenaline sensitivity in mesenteric resistance arteries of streptozotocin-induced diabetic rats. Br. J. Pharmacol. 107 (2), 393–399. 10.1111/j.1476-5381.1992.tb12757.x 1422588 PMC1907852

[B89] Taylor P. D. Wickenden A. D. Mirrlees D. J. Poston L. (1994). Endothelial function in the isolated perfused mesentery and aortae of rats with streptozotocin-induced diabetes: effect of treatment with the aldose reductase inhibitor, ponalrestat. Br. J. Pharmacol. 111 (1), 42–48. 10.1111/j.1476-5381.1994.tb14021.x 8012723 PMC1910054

[B90] Taylor L. E. Gillis E. E. Musall J. B. Baban B. Sullivan J. C. (2018). High-fat diet-induced hypertension is associated with a proinflammatory T cell profile in male and female dahl salt-sensitive rats. Am. J. Physiol. Heart Circ. Physiol. 315 (6), 1713–1723. 10.1152/ajpheart.00389.2018 PMC6336972 30239234

[B91] Teff K. L. Elliott S. S. Tschop M. Kieffer T. J. Rader D. Heiman M. (2004). Dietary fructose reduces circulating insulin and leptin, attenuates postprandial suppression of ghrelin, and increases triglycerides in women. J. Clin. Endocrinol. Metab. 89 (6), 2963–2972. 10.1210/jc.2003-031855 15181085

[B92] Traupe T. Lang M. Goettsch W. Munter K. Morawietz H. Vetter W. (2002). Obesity increases prostanoid-mediated vasoconstriction and vascular thromboxane receptor gene expression. J. Hypertens. 20 (11), 2239–2245. 10.1097/00004872-200211000-00024 12409963

[B93] Tune J. D. Goodwill A. G. Sassoon D. J. Mather K. J. (2017). Cardiovascular consequences of metabolic syndrome. Transl. Res. 183, 57–70. 10.1016/j.trsl.2017.01.001 28130064 PMC5393930

[B94] Vandanmagsar B. Youm Y.-H. Ravussin A. Galgani J. E. Stadler K. Mynatt R. L. (2011). The NLRP3 inflammasome instigates obesity-induced inflammation and insulin resistance. Nat. Med. 17 (2), 179–188. 10.1038/nm.2279 21217695 PMC3076025

[B95] Vanhoutte P. M. Tang E. H. (2008). Endothelium-dependent contractions: when a good guy turns bad! J. Physiol. 586 (22), 5295–5304. 10.1113/jphysiol.2008.161430 18818246 PMC2655387

[B96] Vanhoutte P. M. Feletou M. Taddei S. (2005). Endothelium-dependent contractions in hypertension. Br. J. Pharmacol. 144 (4), 449–458. 10.1038/sj.bjp.0706042 15655530 PMC1576026

[B97] Versari D. Daghini E. Virdis A. Ghiadoni L. Taddei S. (2009). Endothelial dysfunction as a target for prevention of cardiovascular disease. Diabetes Care 32, Suppl 2:S314–21. 10.2337/dc09-S330 PMC2811443 19875572

[B98] Wang B. Chandrasekera P. C. Pippin J. J. (2014). Leptin- and leptin receptor-deficient rodent models: relevance for human type 2 diabetes. Curr. Diabetes Rev. 10 (2), 131–145. 10.2174/1573399810666140508121012 24809394 PMC4082168

[B99] Wang X. DuBois D. C. Sukumaran S. Ayyar V. Jusko W. J. Almon R. R. (2014). Variability in zucker diabetic fatty rats: differences in disease progression in hyperglycemic and normoglycemic animals. Diabetes Metab. Syndr. Obes. 7, 531–541. 10.2147/DMSO.S69891 25419150 PMC4234283

[B100] Wassmann S. Laufs U. Muller K. Konkol C. Ahlbory K. Baumer A. T. (2002). Cellular antioxidant effects of atorvastatin *in vitro* and *in vivo*. Arterioscler. Thromb. Vasc. Biol. 22 (2), 300–305. 10.1161/hq0202.104081 11834532

[B101] Wattanapitayakul S. K. Weinstein D. M. Holycross B. J. Bauer J. A. (2000). Endothelial dysfunction and peroxynitrite formation are early events in angiotensin-induced cardiovascular disorders. FASEB J. 14 (2), 271–278. 10.1096/fasebj.14.2.271 10657983

[B102] Widlansky M. E. Gutterman D. D. (2011). Regulation of endothelial function by mitochondrial reactive oxygen species. Antioxid Redox Signal. 15 (6), 1517–1530. 10.1089/ars.2010.3642 21194353 PMC3151425

[B103] Wind S. Beuerlein K. Armitage M. E. Taye A. Kumar A. H. Janowitz D. (2010). Oxidative stress and endothelial dysfunction in aortas of aged spontaneously hypertensive rats by NOX1/2 is reversed by NADPH oxidase inhibition. Hypertension 56 (3), 490–497. 10.1161/HYPERTENSIONAHA.109.149187 20606112

[B104] Winters B. Mo Z. Brooks-Asplund E. Kim S. Shoukas A. Li D. (2000). Reduction of obesity, as induced by leptin, reverses endothelial dysfunction in obese (Lep(ob)) mice. J. Appl. Physiol. (1985) 89 (6), 2382–2390. 10.1152/jappl.2000.89.6.2382 11090593

[B105] Wong V. Stavar L. Szeto L. Uffelman K. Wang C. H. Fantus I. G. (2006). Atorvastatin induces insulin sensitization in Zucker lean and fatty rats. Atherosclerosis 184 (2), 348–355. 10.1016/j.atherosclerosis.2005.05.009 15998521

[B106] Wong S. K. Chin K. Y. Suhaimi F. H. Fairus A. Ima-Nirwana S. (2016). Animal models of metabolic syndrome: a review. Nutr. Metab. 13, 65 1–12. 10.1186/s12986-016-0123-9 PMC5050917 27708685

[B107] Xu J. Zou M. H. (2009). Molecular insights and therapeutic targets for diabetic endothelial dysfunction. Circulation 120 (13), 1266–1286. 10.1161/CIRCULATIONAHA.108.835223 19786641 PMC2910587

[B108] Yang D. Gluais P. Zhang J. N. Vanhoutte P. M. Feletou M. (2004). Endothelium-dependent contractions to acetylcholine, ATP and the calcium ionophore A 23187 in aortas from spontaneously hypertensive and normotensive rats. Fundam Clin. Pharmacol. 18 (3), 321–326. 10.1111/j.1472-8206.2004.00247.x 15147283

[B109] Yee A M. S. Simpson S. H. McAlister F. A. Tsuyuki R. T. Johnson J. A. (2004). Statin use in Type 2 diabetes mellitus is associated with a delay in starting insulin. Diabetes Med. 21, 962–967. 10.1111/j.1464-5491.2004.01263.x 15317599

[B110] Yokoi N. Hoshino M. Hidaka S. Yoshida E. Beppu M. Hoshikawa R. (2013). A novel rat model of type 2 diabetes: the zucker fatty diabetes mellitus ZFDM rat. J. Diabetes Res. 2013, 103–731. 10.1155/2013/103731 PMC3647587 23671847

[B111] Yoshizumi M. Perrella M. A. Burnett J. C. Jr. Lee M. E. (1993). Tumor necrosis factor downregulates an endothelial nitric oxide synthase mRNA by shortening its half-life. Circ. Res. 73 (1), 205–209. 10.1161/01.RES.73.1.205 7685252

[B112] Zhang Y. H. (2017). Nitric oxide signalling and neuronal nitric oxide synthase in the heart under stress. Res 6, (742) 1–12. 10.12688/f1000research.10128.1 PMC5464233 28649367

[B113] Zhuang H. Lin Y. Yang G. (2007). Effects of 1,25-dihydroxyvitamin D3 on proliferation and differentiation of porcine preadipocyte *in vitro*. Chem. Biol. Interact. 170 (2), 114–123. 10.1016/j.cbi.2007.07.012 17803983

[B114] Zucker L. M. Zucker T. F. (1961). Fatty, a new mutation in the rat. J. Hered. 52 (6), 275–278. 10.1093/oxfordjournals.jhered.a107093

